# Acute Necrotizing Encephalopathy of Childhood: A Report of Two Cases

**DOI:** 10.7759/cureus.103967

**Published:** 2026-02-20

**Authors:** Amal Akammar, Salma Abouchiba, Hajar Ouazzani, Ismail Chaouche, Nizar El Bouardi, Meriem Haloua, Badreddine Alami, Moulay Youssef Alaoui Lamrani, Mustapha Maaroufi, Meryem Boubbou

**Affiliations:** 1 Department of Mother and Child Radiology, Hassan II University Hospital, Sidi Mohamed Ben Abdellah University, Fes, MAR; 2 Department of Radiology, Hassan II University Hospital, Sidi Mohamed Ben Abdellah University, Fes, MAR

**Keywords:** acute necrotizing encephalitis, bilateral thalamic lesions, central nervous system infection, magnetic resonance imaging, rare neurological disorder

## Abstract

Acute necrotizing encephalopathy of childhood (ANEC) is a rare, severe disorder in previously healthy infants and children, associated with high morbidity and mortality. Early recognition is essential for timely management. We report two pediatric cases. A three-month-old female infant presented with hypotonia, feeding refusal, and lethargy; MRI revealed bilateral, symmetrical thalamic lesions with edema. A four-year-old female developed status epilepticus following a febrile illness; imaging showed diffuse, asymmetrical cortical and subcortical lesions, including hemorrhagic changes in the thalami and brainstem. Both patients received aggressive supportive care, immunomodulation with corticosteroids, and empiric antimicrobial therapy. The second patient also required intensive management for status epilepticus. ANEC presents with nonspecific symptoms such as fever, seizures, and altered consciousness. MRI is crucial for diagnosis, demonstrating characteristic lesions that may vary in distribution and severity. Early neuroimaging, prompt supportive care, and awareness of lesion variability are essential for accurate diagnosis and optimizing outcomes in children with ANEC.

## Introduction

Acute necrotizing encephalopathy of childhood (ANEC) is a rare and severe neurological disorder that primarily affects previously healthy infants and children [1]. Although its exact cause is unclear, infections such as *Mycoplasma*, herpes simplex virus (HSV), human herpesvirus-6, and influenza have been implicated. The pathophysiology is characterized by a cytokine storm or hyperimmune response in genetically susceptible individuals [2].

MRI plays a central role in diagnosis, typically revealing bilateral, symmetrical lesions in the thalami, cerebellar white matter, brainstem tegmentum, periventricular white matter, and putamina. These lesions, often accompanied by edema and variable contrast enhancement, help distinguish ANEC from other encephalitic conditions [3]. However, the distribution and symmetry of lesions can vary, and hemorrhagic transformation may occur in severe cases. Management is largely supportive, focusing on symptom control. Corticosteroids and IV immunoglobulins have shown potential benefit in some cases. Despite a historically guarded prognosis, advances in neuroimaging and intensive care have improved patient outcomes [4].

The objective of this report is to illustrate the striking clinical and radiological variability of ANEC through two distinct presentations, highlighting the challenges in diagnosis and the critical role of neuroimaging in predicting prognosis.

## Case presentation

Case 1

A previously healthy three-month-old female infant was admitted to the emergency department with hypotonia, feeding refusal, and absence of crying for the preceding three days. Her parents reported a recent hospitalization for a respiratory infection, but there was no prior history of abnormal behavior or gastrointestinal symptoms. On physical examination, the infant exhibited marked hypotonia and lethargy. Initial laboratory evaluation revealed leukocytosis (WBC 19,000/µL) and an elevated CRP (2.1 mg/dL), with Hb measured at 9.2 g/dL. Extensive microbiological investigations, including Gram stain, bacterial cultures, and PCR for HSV and enteroviruses, were negative, showing no evidence of direct central nervous system infection. Given the concerning clinical presentation, a comprehensive diagnostic workup was initiated (Table 1).

**Table 1:** Case 1: laboratory findings

Parameter	Result	Normal range	Interpretation
WBC	19,000/µL	5,000-15,000/µL	Leukocytosis
Hb	9.2 g/dL	10.5-13.5 g/dL	Anemia
CRP	2.1 mg/dL	<0.5 mg/dL	Elevated

**Figure 1:**
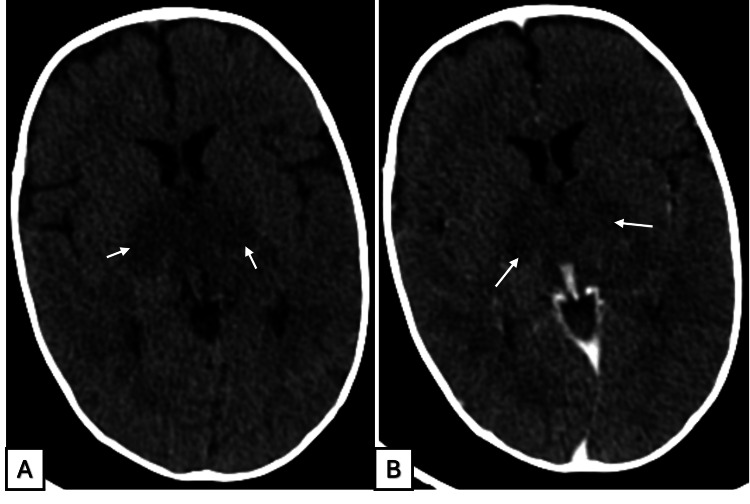
Brain CT findings in ANEC A CT scan without (A) and with contrast enhancement (B) shows bithalamic ill-defined areas of hypodensity, with no visible enhancement. ANEC, acute necrotizing encephalopathy of childhood

**Figure 2:**
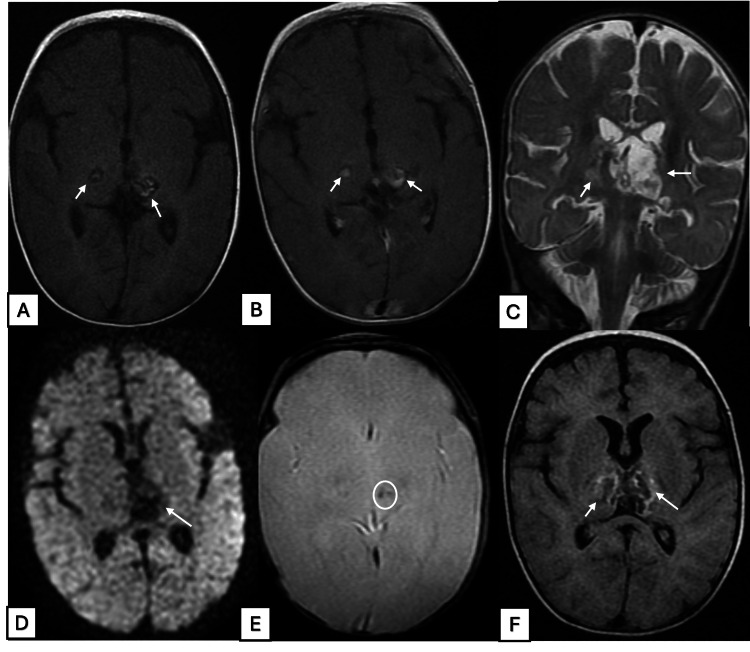
Multimodal MRI findings in ANEC Axial FLAIR and T1-weighted images (A, B) and coronal T2-weighted images (C) show poorly defined hyperintense lesions in the thalami, containing focal areas of hyperintensity (arrows). Axial DWI (D) shows little to no diffusion restriction. T2* gradient-echo imaging (E) demonstrates areas of blooming artifact consistent with hemorrhage (arrowheads). Post-contrast axial T1-weighted images (F) show central annular and heterogeneous enhancement. ANEC, acute necrotizing encephalopathy of childhood; DWI, diffusion-weighted imaging

A non-contrast CT scan demonstrated ill-defined hypodense lesions involving the thalami and midbrain, with minimal contrast enhancement (Figure 1). Subsequent contrast-enhanced MRI revealed multiple symmetrical lesions affecting the thalami and mesencephalic white matter. These lesions appeared hyperintense on T2-weighted and FLAIR sequences, exhibited blooming on T2-weighted gradient echo imaging suggestive of hemorrhagic components, and showed minimal diffusion restriction, with annular contrast enhancement characteristic of acute necrotizing encephalopathy (ANE). No additional abnormal signal intensities were observed in the remainder of the brain parenchyma (Figure 2).

The patient was initiated on empiric broad-spectrum IV antibiotic therapy consisting of ampicillin (200 mg/kg/day divided every six hours) and cefotaxime (200 mg/kg/day divided every six to eight hours) while awaiting confirmatory microbiological testing. Given concern for possible HSV encephalitis, IV acyclovir (20 mg/kg every eight hours) was administered empirically. Supportive care included IV fluids, antiseizure management as clinically indicated, and interventions to address hypotonia, in accordance with institutional pediatric intensive care protocols.

Despite these measures, the clinical course remained complex. The patient survived but exhibited significant neurological sequelae, requiring long-term multidisciplinary rehabilitation. Integration of the clinical features, neuroimaging findings, and exclusion of alternative etiologies led to a diagnosis of ANE. This case highlights the pivotal role of early neuroimaging in identifying the hallmark lesions of ANEC, which is essential for initiating timely and appropriate management.

Case 2

A previously healthy four-year-old female with a history of acute tonsillitis two months earlier was admitted following a generalized tonic-clonic seizure that progressed to status epilepticus. The patient had experienced a high-grade fever of 40°C for 10 days prior to admission, which was treated with antibiotics without clinical improvement. On examination, the child had a Glasgow Coma Scale score of 9. Laboratory investigations revealed no anemia (Hb 12 g/dL), leukocytosis (WBC 31,000/µL), and an elevated CRP (16 mg/L). CSF analysis, performed during the initial diagnostic workup, was within normal limits (Table 2).

**Table 2:** Case 2: laboratory findings

Parameter	Result	Normal range	Interpretation
WBC	31,000/µL	5,000-15,000/µL	Leukocytosis
Hb	12 g/dL	10.5-13.5 g/dL	No abnormalities detected
CRP	16 mg/L	<10 mg/L	Elevated, consistent with acute inflammation or infection

**Figure 3:**
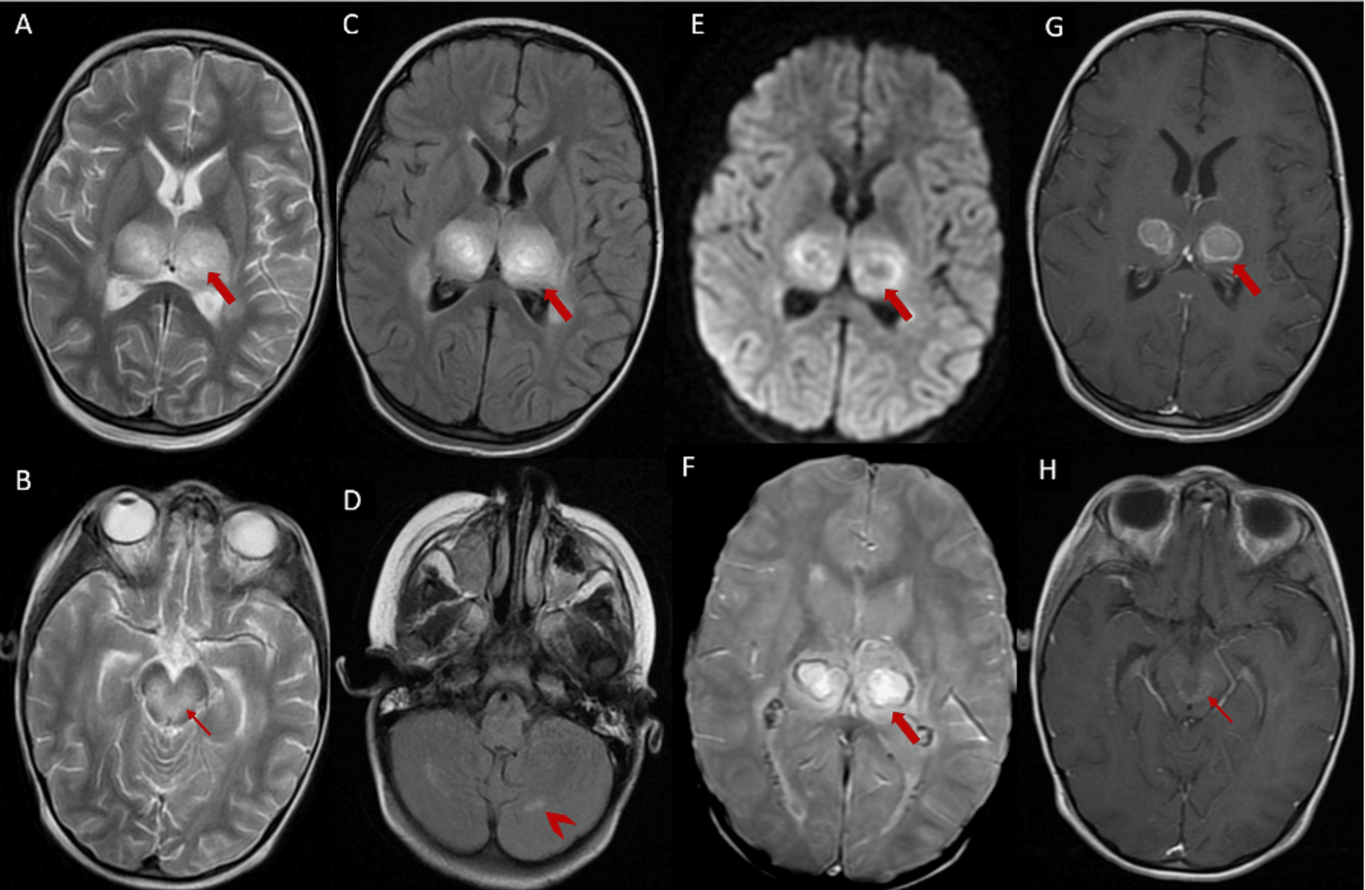
Axial multisequence MRI demonstrating necrotizing encephalopathy Axial T2-weighted images (A, B) and axial FLAIR images (C) show bithalamic lesions (thick arrows), involving the medial parts of the midbrain and brainstem tegmentum (thin arrow) and cerebellar white matter (arrowhead), with spontaneous hyperintensity on T2 and FLAIR, most pronounced at the thalami. Susceptibility-weighted imaging (F) shows a peripheral signal void consistent with hemorrhagic stigmata. Post-contrast axial T1-weighted images (G, H) show mild enhancement after contrast administration. The lesions are surrounded by a FLAIR hyperintense zone associated with peri-lesional edema.

A contrast-enhanced CT scan demonstrated hypodense lesions involving the thalami and mesencephalic-pontine regions. A subsequent MRI confirmed these findings and revealed symmetrical hemorrhagic lesions in the bithalamic region and brainstem, evident as blooming on T2-weighted gradient-echo sequences with annular contrast enhancement. Additionally, hyperintensities on FLAIR sequences were noted affecting the corpus callosum and both supratentorial and infratentorial white matter (Figure 3). These radiologic features were consistent with ANE.

The diagnosis of ANEC was established based on the characteristic neuroimaging findings. The patient was treated with empiric IV broad-spectrum antibiotics, including ampicillin (200 mg/kg/day divided every six hours) and cefotaxime (200 mg/kg/day divided every six to eight hours), as well as antiviral therapy with IV acyclovir (20 mg/kg every eight hours). To address the inflammatory component and mitigate presumed inflammatory brain edema, systemic corticosteroid therapy with IV dexamethasone (0.15 mg/kg every six hours) was administered.

Despite aggressive medical management, the clinical course deteriorated rapidly, leading to acute oxygen desaturation and subsequent cardiopulmonary arrest. Resuscitation efforts were unsuccessful, and the patient died.

## Discussion

ANEC is a rapidly progressive and devastating neurological disorder characterized by a highly variable clinical course and a challenging diagnostic process. Although rare, increasing recognition of its diverse presentations, ranging from survival with neurological sequelae to rapid fatality, highlights the need for early clinical suspicion and prompt neuroimaging [4]. Originally described in Japan and Taiwan, sporadic cases have since been documented worldwide, indicating a wider geographic distribution than previously recognized [5]. Our two cases illustrate this necessity by demonstrating how similar diagnostic criteria can lead to vastly different clinical trajectories.

This rapidly progressive condition typically presents with nonspecific symptoms, including high-grade fever, seizures, and altered neurological status. Approximately 40% of patients with ANEC experience convulsions, 28% present with decreased consciousness, and 20% report vomiting [6]. CSF studies often show elevated protein levels, although pleocytosis is usually absent [7]. In the chronic phase, affected children may develop motor impairments such as intention tremor, ataxia, speech difficulties, choreoathetosis, and spasticity. Hemiparesis and abnormal extraocular movements have also been reported as long-term complications [2].

Radiologically, ANEC is characterized by multifocal, symmetrical lesions affecting both gray and white matter. MRI remains the imaging modality of choice for identifying these lesions, which most commonly involve the thalami, brainstem, cerebral white matter, and cerebellum [1]. Conventional MRI consistently demonstrates bilateral, symmetrical thalamic involvement. Beyond the thalami, additional regions may be affected, including the basal ganglia (~13% of cases), brainstem (80%), cerebellar white matter (33%), and cerebral white matter (73%) [3]. On MRI, lesions are typically hypointense on T1-weighted sequences and hyperintense on T2-weighted and FLAIR sequences. Diffusion-weighted imaging (DWI) often reveals cytotoxic edema with mass effect, manifesting as hyperintensity on DWI and corresponding hypointensity on ADC maps [8]. Hemorrhagic changes, cavitation, and surrounding edema are frequently observed. Susceptibility-weighted imaging can identify hemosiderin deposits in the thalami, brainstem, and cerebellum that may be overlooked on conventional sequences [8]. In our report, identification of these hemorrhagic components through “blooming” on T2 sequences was crucial to confirm the necrotizing pathology in both patients. Gadolinium-enhanced MRI is particularly useful for early diagnosis, as it can detect lesions not visible on standard sequences. These findings support the hypothesis that blood-brain barrier disruption is a key early event, likely triggered by a systemic cytokine storm. This immune-mediated mechanism leads to the characteristic vasogenic edema and neuroinflammation observed in ANEC pathogenesis [5,9].

In terms of differential diagnosis, conditions affecting the deep gray matter, such as toxic encephalopathy, hemolytic uremic syndrome, hemorrhagic shock, and metabolic encephalopathies, should be considered. Clinical and biochemical investigations are essential to exclude metabolic disorders. Acute disseminated encephalomyelitis (ADEM) and Reye’s syndrome also represent important differentials. While ADEM often presents with bilateral but asymmetric involvement of white and gray matter, Reye’s syndrome is typically associated with prior aspirin use following viral infection and is characterized by hepatic and renal dysfunction [10].

Management of ANEC is primarily supportive, including hydration, corticosteroids, and anticonvulsants. Empiric antiviral therapy is sometimes administered but discontinued if viral serologies, such as for HSV, are negative. Despite intensive care, ANEC remains a rapidly progressive and severe disease with variable outcomes. Mortality is reported around 30%, and complete recovery occurs in fewer than 10% of cases. Long-term neurological sequelae are common among survivors [11,12].

These cases provide critical insights into the radiological spectrum of ANEC and its clinical implications. In Case 1, neuroimaging revealed prominent bilateral thalamic lesions with marked edema; despite their severity, the relatively localized distribution of these lesions was consistent with survival, though with neurological sequelae. In contrast, Case 2 exhibited a much broader radiological involvement, including the thalami, brainstem, corpus callosum, and supratentorial and infratentorial white matter. This extensive involvement serves as a key prognostic indicator, directly correlating with the rapid clinical deterioration and fatal outcome observed.

Our findings highlight that, while certain patterns are typical, the manifestations of ANEC are highly heterogeneous and carry significant prognostic value. Awareness of this variability is crucial for clinicians, as careful interpretation of MRI findings, specifically the extent of extra-thalamic involvement, is essential to support accurate diagnosis, predict clinical outcomes, and guide individualized management strategies.

## Conclusions

These cases illustrate that ANEC is not a uniform entity but a spectrum with striking clinical and radiological variability. Our report showcases two distinct presentations in which similar initial features led to vastly different trajectories, ranging from survival with significant neurological sequelae to rapid fatality. This diversity underscores that careful interpretation of necrotic patterns on MRI is not only diagnostic but also serves as a critical prognostic tool, directly reflecting the disease severity.

Furthermore, while management remains primarily supportive, the potential role of early immunomodulation, such as high-dose corticosteroids, should be incorporated into aggressive treatment strategies. Clinicians should maintain a high index of suspicion and prioritize early neuroimaging, as prompt recognition of these variable signatures provides the only window of opportunity to optimize outcomes in this unpredictable disorder.
